# Prognostic nomogram for 30-day mortality of deep vein thrombosis patients in intensive care unit

**DOI:** 10.1186/s12872-020-01823-4

**Published:** 2021-01-06

**Authors:** Runnan Shen, Ming Gao, Yangu Tao, Qinchang Chen, Guitao Wu, Xushun Guo, Zuqi Xia, Guochang You, Zilin Hong, Kai Huang

**Affiliations:** 1 Department of Cardiovascular Surgery, Sun Yat-Sen Memorial Hospital, Sun Yat-Sen University, No. 33, Yingfeng Road, Haizhu District, Guangzhou, 510000 Guangdong Province China; 2 Department of Radiology, Sun Yat-Sen Memorial Hospital, Sun Yat-Sen University, No. 33, Yingfeng Road, Haizhu District, Guangzhou, 510000 Guangdong Province China; 3 Department of Traditional Chinese Medicine, Sun Yat-Sen Memorial Hospital, Sun Yat-Sen University, No. 33, Yingfeng Road, Haizhu District, Guangzhou, 510000 Guangdong Province China; 4 The First Affiliated Hospital, Sun Yat-Sen University, No. 58, Zhongshan Rd.2, Guangzhou, 510080 Guangdong Province China; 5 Zhongshan School of Medicine, Sun Yat-Sen University, No. 58, Zhongshan Rd.2, Guangzhou, 510080 Guangdong Province China

**Keywords:** Prognosis, Nomogram, Deep vein thrombosis, Intensive care unit

## Abstract

**Background:**

We aimed to use the Medical Information Mart for Intensive Care III database to build a nomogram to identify 30-day mortality risk of deep vein thrombosis (DVT) patients in intensive care unit (ICU).

**Methods:**

Stepwise logistic regression and logistic regression with least absolute shrinkage and selection operator (LASSO) were used to fit two prediction models. Bootstrap method was used to perform internal validation.

**Results:**

We obtained baseline data of 535 DVT patients, 91 (17%) of whom died within 30 days. The discriminations of two new models were better than traditional scores. Compared with simplified acute physiology score II (SAPSII), the predictive abilities of two new models were improved (Net reclassification improvement [NRI] > 0; Integrated discrimination improvement [IDI] > 0; *P* < 0.05). The Brier scores of two new models in training set were 0.091 and 0.108. After internal validation, corrected area under the curves for two models were 0.850 and 0.830, while corrected Brier scores were 0.108 and 0.114. The more concise model was chosen to make the nomogram.

**Conclusions:**

The nomogram developed by logistic regression with LASSO model can provide an accurate prognosis for DVT patients in ICU.

## Background

Patients in ICU are at a high risk of developing DVT. Among patients who do not receive anticoagulation therapy in ICU, the prevalence of DVT is between 10 and 80% [1]. According to the classical Virchow’s triad, the stagnation of blood flow in patients within ICU due to long-term bedridden is an important factor that makes them more likely to suffer from DVT [2, 3]. In addition, endothelial damage caused by mechanical ventilation, catheterization and infection also increases the risk [2]. DVT is one kind of venous thromboembolism (VTE), which can cause acute pulmonary embolism (PE) if thrombi in lower extremity falls and flows back to the lung [4, 5]. Current guidelines recommend anticoagulation or thrombolytic therapy for patients with DVT found in ICU [5]. However, since patients in ICU are often complicated with multiple organ dysfunction or renal failure, anticoagulation or thrombolysis may cause major bleeding and lead to death [6, 7]. Therefore, DVT in ICU is in urgent need of attention. Patients in ICU are characterized by dynamic changes in thrombosis threshold and bleeding risk from day to day [2], which causes fluctuations in the mortality risk and brings challenges for doctors to make clinical decisions. The establishment of relevant scoring or prediction models can assist doctors in ICU to make important decisions in a short time. However, there is lack of a scoring model for predicting the risk of mortality of DVT patients in ICU. The existing scores associated with DVT, like Wells, Padua and Caprini, are not suitable for critically ill patients [8–10], and also can’t predict future mortality [11–13]. We hoped to fully include the initial admission information of DVT patients in ICU, consisting of general status, comorbidity information and important laboratory indicators to make a concise prediction model and present as nomogram for predicting the risk of 30-day mortality.

Nomogram is a graphical calculation tool designed to perform complex calculations quickly. Because of the trend of precision medicine and the aim to reach the simplicity of clinical decision-making, nomogram is widely used in medical treatment, especially in clinical predictive modeling [14, 15]. We also presented our prediction model as nomogram in this study in order to promote the decision-making ability for DVT patients in ICU in the future.

## Methods

### Data retrieval

The data used in this study was obtained from Medical Information Mart for Intensive Care III (MIMIC-III) database. The MIMIC-III database integrates information on more than 50,000 ICU hospitalized patients from Beth Israel Deaconess Medical Center in Boston, Massachusetts from 2001 to 2012.[16]. MIMIC database uses international classification of diseases (ICD)-9 diagnostic code to define the patient’s condition, which we used to select the baseline data of patients diagnosed as lower extremity DVT (ICD-9 diagnostic code including 45340, 45341, 45342, 45350, 45351 and 45352). We extracted the admission data and clinical outcomes of all patients diagnosed as DVT. The admission data included general condition, comorbidity information, laboratory indicators and severity scores. The clinical outcome was defined as all-cause death within 30 days after admission. Variables with missing data of more than 30% were excluded. In the general admission condition of patients, heart rate, systolic blood pressure, diastolic blood pressure, respiratory rate, body temperature, percutaneous oxygen saturation (spO2) and blood glucose were all the average values of the data collected on first day of ICU admission. In laboratory indicators, red blood cell volume distribution width (RDW), lymphocyte, monocyte, neutrophil, hematocrit, white blood cell and platelet count, blood sodium, blood potassium, serum anion gap, serum bicarbonate, serum chloride, blood urea nitrogen, serum creatinine, albumin, hemoglobin, serum lactate, partial thromboplastin time (PTT) and prothrombin time (PT) were all defined as the average values of data collected on the first day of admission or the earliest day after admission if detection delayed. Neutrophil-to-lymphocyte ratio (NLR) and lymphocyte-to-monocyte ratio (LMR) were also accessed using laboratory data detected at the first time. The comorbidity information and severity scores of each patient were collected using the code on github [17] to establish materialized views and extract relevant information on the PostgreSQL software. Comorbidity information was the ICD-9 diagnosis of patient who was admitted to hospital at the same time.

### Statistical analysis

In the baseline data of patients, continuous variables with normal distribution would be represented by mean with standard deviation (SD), compared with t-test. Continuous variables with abnormal distribution would be represented by median and interquartile range, compared with Wilcoxon rank-sum test. Categorical variables would be represented by frequency and percentage, compared with chi-square test. We used general conditions, comorbidity information and laboratory indicators from patients’ baseline data as candidate variables to develop new models. First of all, we transformed the variables including RDW, monocyte, creatinine, lactate, NLR and LMR which with abnormal distribution to new variables that conformed to normal distribution (we used a stable and large value of 50 minus each value of RDW to get a new variable, and monocyte, creatinine, lactate, NLR and LMR were transformed using the logarithm of 10 to construct new variables). For some variables that contained outliers (glucose and anion gap) and would affect the stability of the final model, we truncated them at 1% and 99%. Then we performed single imputation for the whole dataset based on the complete conditional specification and used predictive mean matching method to fill the missing value. Each incomplete variable was estimated by an independent model to ensure the validity of the imputation results. This process was implemented using the “mice” package in R software (Version: 3.6.1) [18]. For the convenience of using the model in clinic, we chose to convert all continuous variables into categorical variables. We used restricted cubic splines to detect the linear relationship between continuous variables and clinical outcome. Group transformations according to the analysis of restricted cubic splines were carried out for continuous variables that were not linear with the clinical outcome [19]. For the continuous variables that had a linear relationship with the outcome, the best categorical cut-off value of them would be determined by clinical significance or the analysis of the receiver operating characteristic (ROC) curves where the Youden index reached the maximum.

### Model building

Firstly, we used stepwise logistic regression to fit the model with all candidate variables and the outcome of 30-day mortality, and selected the model with the smallest akaike information criterion (AIC). Considering the convenience of clinical application, we then used LASSO to screen all candidate variables. LASSO can help to simplify the model by increasing penalty coefficients λ to compress the estimate of each variable [20]. After that, less variables were selected according to LASSO results and clinical significance. Multivariate logistic regression was then used to fit a more concise model. After building two new models using stepwise logistic regression and logistic regression with L1 regularization (i.e. LASSO), these two new models were used to calculate the discrimination and calibration in the original training set. Discrimination was measured by area under the curves (AUC) and calibration was measured by Brier score. The Brier score was calculated as the following formula:

$$\mathrm{Brier\, Score}=\frac{1}{N}\sum_{t=1}^{N}{({f}_{t}-{o}_{t})}^{2}$$

In the formula, N represents the total number of predictions, ${f}_{t}$ represents the actual results and ${o}_{t}$ represents the prediction probability of the model. Then AUC, NRI and IDI were used as indicators to compare predictive abilities of two new models and traditional scoring models. Bootstrap was used to validate the two new models internally and the number of repetitions was set as 100 [21]. Finally, for the convenience of clinical application, we made a nomogram according to the model fitted by logistic regression with LASSO. The above data processing was done in R software (Version: 3.6.1).

## Results

**Fig. 1:**
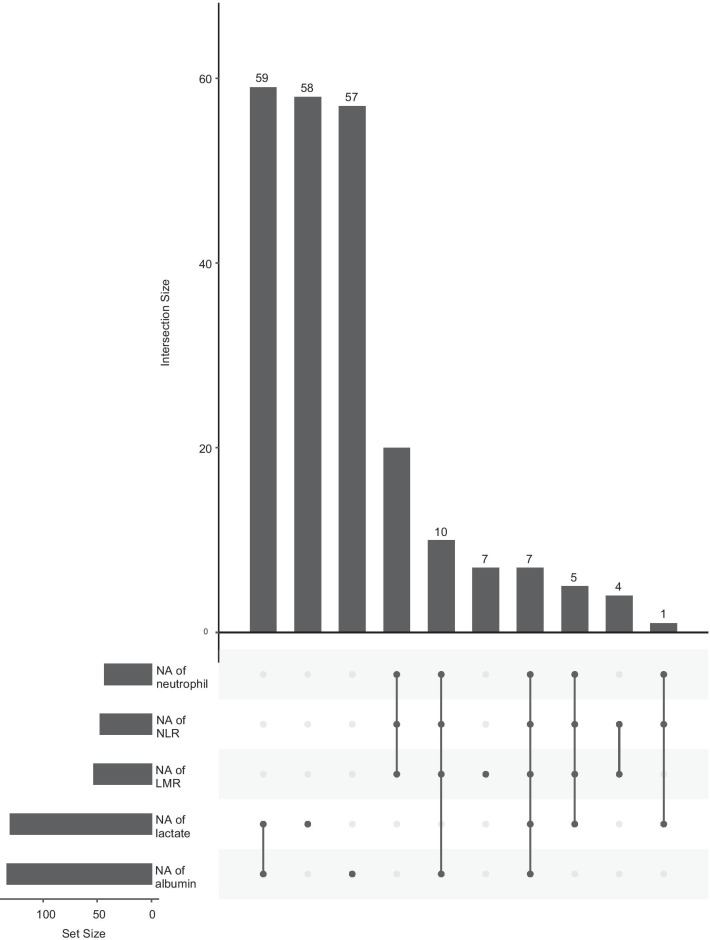
Situation of data missing. The histograms on the lower left represent the number of missing values of the top five variables which have most missing data, while the vertical line on the lower right and the histograms above represent the situation of common deletions. Among them, lactate and albumin are more deficient, missing data of which are both more than 20% of themselves. *NA* not available, *NLR* neutrophil-to-lymphocyte ratio, *LMR* lymphocyte-to-monocyte ratio

**Fig. 2:**
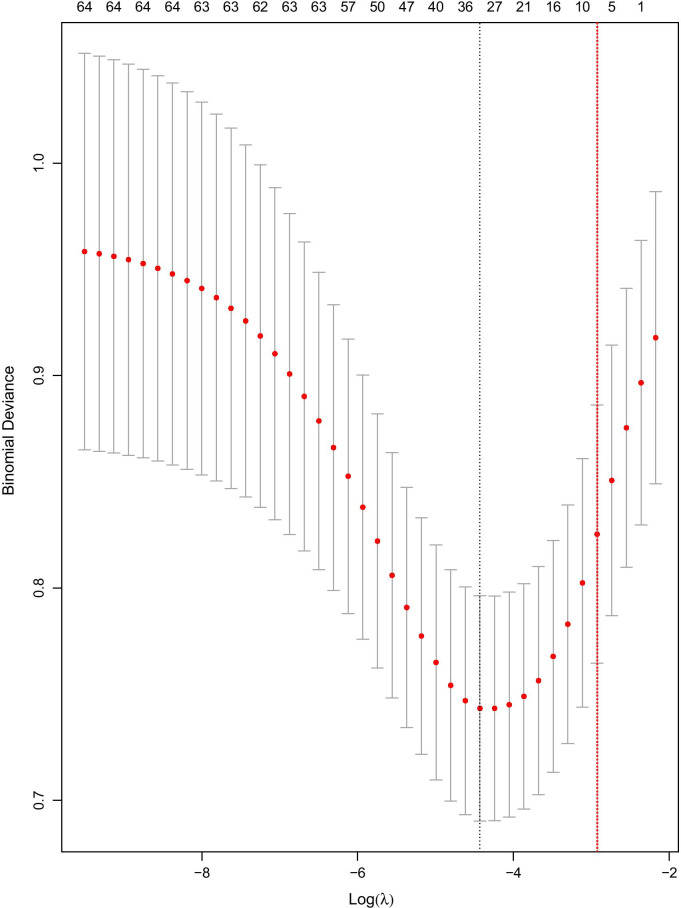
Cross-validation of logistic regression with LASSO. Lower abscissa represents the continuous increase of penalty coefficient λ, and the upper represents the continuous decrease of variables in the model from left to right. The ordinate represents corresponding binomial deviation (or minimum mean cross-validated error) of each model. The black dotted line represents the model with the least binomial deviation while the red one represents the model including eight variables we selected in this study. Line segment of each model represents 95% CI of binomial deviation. *LASSO* least absolute shrinkage and selection operator, *CI* confidence interval

**Fig. 3:**
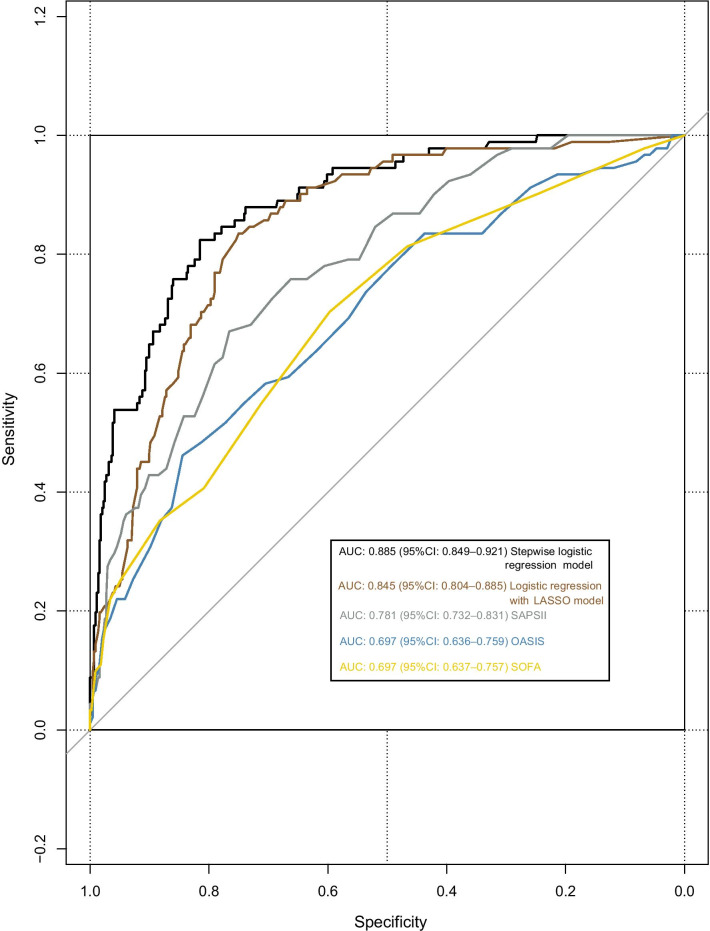
ROC curves of two new models and traditional scores. *ROC* receiver operating characteristic, *AUC* area under the curve, *CI* confidence interval, *LASSO* least absolute shrinkage and selection operator, *SAPSII* simplified acute physiology score II, *OASIS* oxford acute severity of illness score, *SOFA* sequential organ failure assessment

**Fig. 4:**
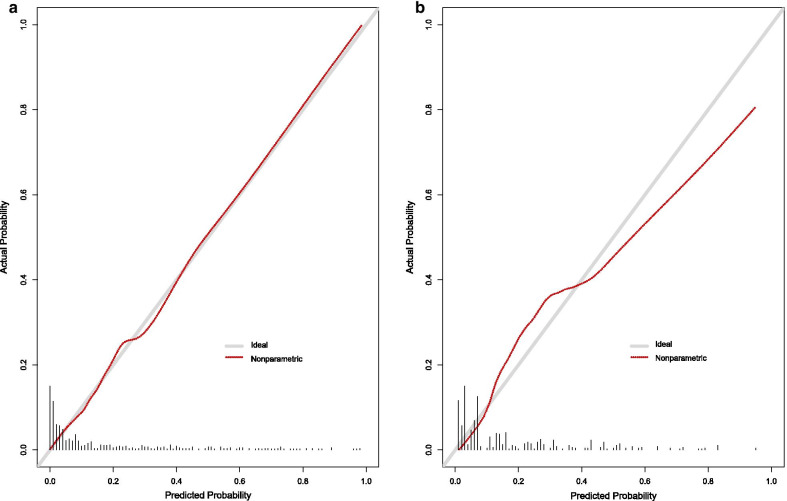
Calibration curves of two new models. **a** Represents the calibration curve of stepwise logistic regression model, **b** represents the calibration curve of logistic regression with LASSO model. Calibration curve shows the mean predicted probability of outcome against the observed proportion of clinical outcomes. *LASSO* least absolute shrinkage and selection operator

**Fig. 5:**
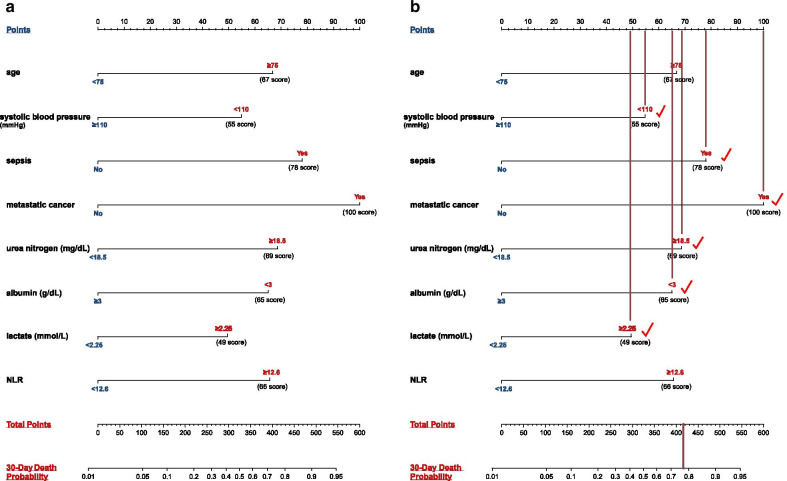
The nomogram obtained from the logistic regression with LASSO model. **a** If a patient meets the target requirement in each variable, he/she will be given a score according to the points above (considering that the nomogram is difficult to calculate with naked eyes, the score that each indicator get has been pointed out). The total score obtained by adding up the scores of all indicators can be compared with the risk of 30-day death probability below. **b** A simple case analysis: a 64 years old female got deep vein thrombosis when admitted to intensive care unit. She had complications of sepsis and metastatic cancer. Her systolic blood pressure was 107 mmHg. After laboratory test, her blood urea nitrogen was 32 mg/dL, albumin was 2.5 g/dL, lactate was 3.2 mmol/L and NLR was 10.7. According to our nomogram, she finally got 416 score and a 30-day death probability that higher than 0.75. *LASSO* least absolute shrinkage and selection operator, *NLR* neutrophil-to-lymphocyte ratio

Of all 535 DVT patients enrolled in this study, 91 patients died within 30 days after admission. In the previous data cleaning, we removed variables that had more than 30% missing data, including most of the variables for blood gas analysis. In remaining variables, missing data of lactate and albumin were more than 20%; Missing data of other variables were no more than 10%. The top five variables with most missing data were albumin, lactate, LMR, NLR and neutrophils. Clinical outcome had none missing data. Figure 1 shows the situation of missing values. The statistical description and univariate analysis of baseline data of patients is shown in Table 1. After classifying all the continuous variables into categorical variables, we incorporated the general condition, comorbidity information and laboratory indicators of all 535 patients’ baseline data into the stepwise logistic regression model. Among them, the location of DVT in patients was excluded because of its uncertain classification, and monocyte was excluded because of poor prediction of outcome. The specific classification of variables is shown in Additional file 1: Table S1. Finally, AIC of the stepwise logistic regression model was 356.75, and 18 variables were included in the model. In order to further simplify the model, we used LASSO to screen all the candidate variables of 535 patients. Then tenfold cross validation for logistic regression was conducted to help select the suitable penalty coefficient λ. The relationship between penalty coefficient λ and variables remaining in the model is shown in Fig. 2. Finally, the penalty coefficient λ as 0.0536 and a model consisting of 8 variables were chosen. The selected 8 variables were then put into multivariable logistic regression to fit the model. The AIC of logistic regression with LASSO model was 382.96. The variables and their coefficients in stepwise logistic regression model and logistic regression with LASSO model are shown in Table 2. ROC curves were then conducted to show the predictive abilities of two new models, SAPSII, oxford acute severity of illness score (OASIS) and sequential organ failure assessment (SOFA) on the clinical outcome of DVT patients. The results showed that the AUC of stepwise logistic regression was the best, which was 0.885 (95% confidence intervals [CI]: 0.849–0.921). The second was the logistic regression with LASSO model, AUC of which was 0.845 (95% CI: 0.804–0.885). Among the three severity scores, AUC of SAPSII took the highest, which was 0.781 (95% CI: 0.732–0.831). The ROC curves are shown in Fig. 3. Then, we evaluated the calibration of stepwise logistic regression model and logistic regression with LASSO model in the original training set. The results showed that the Brier score of stepwise logistic regression model was 0.091, and the Brier score of logistic regression with LASSO model was 0.108. Calibration curves of two new models are shown in Fig. 4. Then NRI and IDI were used to evaluate the improvement of predictive abilities of two new models compared with SAPSII. The classification nodes of risk of mortality were set as 0.2, 0.6 and 0.8. The results showed that the prediction or classification abilities of the stepwise logistic regression model and logistic regression with LASSO model were both better than SAPSII (NRI [categorical and continuous] both > 0; IDI both > 0), and there were statistical significances. Related results about NRI and IDI are shown in Table 3. After using bootstrap for internal validation, the corrected AUC and Brier score of the stepwise logistic regression were 0.850 and 0.105, respectively. And for logistic regression with LASSO model, the results were 0.830 and 0.114, respectively. Related results about internal validation of two new models are shown in Table 4. Although the prediction ability of stepwise logistic regression model was better than that of logistic regression with LASSO model in further analysis (NRI [Continuous] = 0.658 [95% CI 0.442–0.873]; IDI = 0.113 [95% CI 0.071–0.155]; *P* < 0.001), considering the convenience of clinical application of the model and the good enough prediction ability of logistic regression with LASSO model, logistic regression with LASSO model was chosen to make a nomogram. Finally, the converted new variables (transformed from lactate and NLR) in logistic regression with LASSO model were converted back to the original data form to facilitate clinical application. The final nomogram is shown in Fig. 5, which contains 8 variables.

**Table 1:** Baseline data of DVT patients

Variables	Survival group	Death group	*P* value
	444	91	
General condition			
Age	64.36 (± 15.43)	71.51 (± 14.23)	< 0.001
Male	278 (62.6%)	53 (58.2%)	0.507
Admission type			0.821
Emergency	424 (95.5%)	88 (96.7%)	
Elective	19 (4.3%)	3 (3.3%)	
Urgent	1 (0.2%)	0 (0.0%)	
Position of DVT			0.079
Unspecified	70 (15.8%)	23 (25.3%)	
Proximal	273 (61.5%)	52 (57.1%)	
Distal	101 (22.7%)	16 (17.6%)	
Acute DVT	420 (94.6%)	89 (97.8%)	0.304
Heartrate (bpm)	88.38 (± 15.72)	92.40 (± 19.41)	0.036
Systolic blood pressure (mmHg)	119.51 (± 15.14)	109.95 (± 14.15)	< 0.001
Diastolic blood pressure (mmHg)	64.69 (± 10.63)	58.60 (± 10.30)	< 0.001
Respiratory rate (1/min)	19.67 (± 4.08)	21.33 (± 4.81)	0.001
Temperature ( °C)	36.80 (± 0.61)	36.53 (± 0.59)	< 0.001
spO2 (%)	97.16 (± 1.76)	96.75 (± 2.88)	0.075
Glucose (mg/dl)	136.15 (± 40.16)	142.66 (± 48.16)	0.185
Comorbidity			
Infection	253 (57.0%)	70 (76.9%)	0.001
Sepsis	39 (8.8%)	33 (36.3%)	< 0.001
Organ dysfunction	222 (50.0%)	70 (76.9%)	< 0.001
Congestive heart failure	79 (17.8%)	26 (28.6%)	0.027
Cardiac arrhythmias	92 (20.7%)	34 (37.4%)	0.001
Valvular disease	22 (5.0%)	5 (5.5%)	1
Pulmonary circulation disorder	104 (23.4%)	25 (27.5%)	0.491
Peripheral vascular disease	33 (7.4%)	11 (12.1%)	0.207
Hypertension	44 (9.9%)	19 (20.9%)	0.005
Paralysis	33 (7.4%)	5 (5.5%)	0.666
Neurological disease	63 (14.2%)	10 (11.0%)	0.521
Chronic pulmonary disease	72 (16.2%)	29 (31.9%)	0.001
Diabetes	107 (24.1%)	21 (23.1%)	0.942
Hypothyroidism	46 (10.4%)	11 (12.1%)	0.764
Renal failure	51 (11.5%)	22 (24.2%)	0.002
Liver disease	22 (5.0%)	5 (5.5%)	1
Lymphoma	5 (1.1%)	4 (4.4%)	0.078
Metastatic cancer	39 (8.8%)	20 (22.0%)	0.001
Solid tumor	20 (4.5%)	6 (6.6%)	0.564
Obesity	32 (7.2%)	10 (11.0%)	0.313
Blood loss anemia	12 (2.7%)	5 (5.5%)	0.291
Alcohol abuse	32 (7.2%)	3 (3.3%)	0.254
Depression	48 (10.8%)	7 (7.7%)	0.482
Laboratory Indicators			
RDW (%)	15.00 (14.00, 16.00) ^a^	16.00 (14.50, 17.00) ^a^	< 0.001
Lymphocyte (%)	13.67 (± 9.04)	9.26 (± 7.22)	< 0.001
Monocyte (%)	4.00 (3.00, 6.00) ^a^	4.00 (3.00, 5.75) ^a^	0.252
Neutrophil (%)	78.22 (± 13.16)	81.72 (± 12.90)	0.025
Hematocrit (%)	34.53 (± 6.81)	32.97 (± 6.77)	0.047
White blood cell count (K/μL)	11.58 (± 5.96)	14.09 (± 8.29)	0.001
Platelet count (K/μL)	254.05 (± 127.01)	273.23(± 161.47)	0.213
Potassium (mEq/L)	4.27 (± 0.83)	4.34 (± 0.74)	0.472
Sodium (mEq/L)	138.33 (± 4.89)	137.47 (± 5.88)	0.145
Anion gap (mEq/L)	15.24 (± 3.76)	15.44 (± 3.94)	0.653
Bicarbonate (mEq/L)	24.29 (± 4.19)	23.77 (± 5.12)	0.303
Chloride (mEq/L)	103.09 (± 6.38)	102.80 (± 6.79)	0.697
Blood urea nitrogen (mg/dL)	24.06 (± 17.17)	34.06 (± 22.25)	< 0.001
Creatinine (mg/dL)	1.00 (0.80, 1.30) ^a^	1.20 (0.70, 1.95) ^a^	0.066
Albumin (g/dL)	3.14 (± 0.66)	2.70 (± 0.71)	< 0.001
Hemoglobin (g/dL)	11.66 (± 2.40)	10.95 (± 2.34)	0.01
Lactate (mmol/L)	1.70 (1.30, 2.60)^a^	2.30 (1.50, 3.00)^a^	0.008
PTT (s)	37.90 (± 26.91)	37.35 (± 24.97)	0.857
PT (s)	16.84 (± 12.45)	20.31 (± 20.61)	0.034
NLR	7.12 (3.81, 12.00)^a^	12.29 (5.36, 19.96)^a^	< 0.001
LMR	2.79 (1.69, 4.32)^a^	2.11 (1.08, 3.26)^a^	0.001
Severity scores			
OASIS	31.20 (± 8.29)	37.84 (± 9.84)	< 0.001
SAPSII	33.43 (± 12.54)	48.66 (± 15.30)	< 0.001
SOFA	3.44 (± 2.67)	5.97 (± 4.03)	< 0.001

*DVT* deep venous thrombosis, *spO2* percutaneous oxygen saturation, *RDW* red blood cell volume distribution width, *PTT* partial thromboplastin time, *PT* prothrombin time, *NLR* neutrophil-to-lymphocyte ratio, *LMR* lymphocyte-to-monocyte ratio, *OASIS* oxford acute severity of illness score, *SAPSII* simplified acute physiology score II, *SOFA* sequential organ failure assessment

^a^Continuous variables that do not conform to normal distribution were represented by median and interquartile range

**Table 2:** Variables and coefficients included in stepwise logistic regression model and logistic regression with LASSO model

Variables	Stepwise logistic regression			Logistic regression with LASSO		
	Estimate	SE	*P* value	Estimate	SE	*P* value
Intercept	− 5.964	1.158	–	− 2.716	0.379	–
General Condition						
Age						
≥ 75	1.424	0.320	< 0.001	0.887	0.282	0.002
Systolic blood pressure (mmHg)						
≥ 110	− 0.748	0.319	0.019	-0.727	0.281	0.010
Respiratory rate (1/min)						
≥ 30	1.172	0.729	0.108	–	–	–
Acute DVT						
Yes	2.333	0.923	0.012	–	–	–
Comorbidity						
Sepsis						
Yes	1.083	0.370	0.003	1.036	0.325	0.001
Pulmonary circulation disorder						
Yes	0.673	0.336	0.045	–	–	–
Neurological disease						
Yes	− 0.979	0.491	0.046	–	–	–
Chronic pulmonary disease						
Yes	1.167	0.346	< 0.001	–	–	–
Lymphoma						
Yes	1.861	0.827	0.024	–	–	–
Metastatic cancer						
Yes	1.782	0.414	< 0.001	1.328	0.366	< 0.001
Obesity						
Yes	1.180	0.553	0.033	–	–	–
Laboratory Indicators						
50-RDW (%)						
≥ 34.5	− 0.646	0.295	0.028	–	–	–
Lymphocyte (%)						
≥ 8.5	− 0.749	0.307	0.015	–	–	–
Anion gap (mEq/L)						
≥ 16	− 1.007	0.392	0.010	–	–	–
14–16	− 0.427	0.401	0.287	–	–	–
Blood urea nitrogen (mg/dL)						
≥ 18.5	1.292	0.378	< 0.001	0.911	0.304	0.003
Log_10_(creatinine (mg/dL))						
≤ − 0.1	1.205	0.394	0.002	–	–	–
≥ 0.2	0.724	0.385	0.060	–	–	–
Albumin (g/dL)						
≥ 3	–	–	–	− 0.864	0.287	0.003
Log_10_(lactate (mmol/L))						
≥ log_10_(2.25)	0.927	0.311	0.003	0.657	0.270	0.015
PT (s)						
≥ 14.5	0.809	0.308	0.009	–	–	–
Log_10_(NLR)						
≥ 1.1	–	–	–	0.872	0.276	0.002

*LASSO* least absolute shrinkage and selection operator, *SE* standard error, *DVT* deep vein thrombosis, *RDW* red blood cell volume distribution width, *PT* prothrombin time, *NLR* neutrophil-to-lymphocyte ratio

**Table 3:** Improvement in prediction abilities of two new models compared with SAPSII score

	Stepwise logistic regression^a^	*P* value	Logistic regression with LASSO^a^	*P* value
NRI (categorical) (95% CI)	0.352 (0.187–0.517)	< 0.001	0.167 (0.006–0.328)	0.042
NRI (continuous) (95% CI)	0.792 (0.580–1.004)	< 0.001	0.422 (0.200–0.643)	< 0.001
IDI (95% CI)	0.186 (0.119–0.254)	< 0.001	0.073 (0.016–0.131)	0.012
AUC (95% CI)	0.885 (0.849–0.921)	< 0.001	0.845 (0.804–0.885)	0.021

*SAPSII* simplified acute physiology score II, *LASSO* least absolute shrinkage and selection operator, *NRI* net reclassification improvement, *IDI* integrated discrimination improvement, *AUC* area under the curve, *CI* confidence interval

^a^Compared with SAPSII score

**Table 4:** Corrected performance of two new models after internal validation

	Stepwise logistic regression			Logistic regression with LASSO		
	Original	Optimism	Corrected	Original	Optimism	Corrected
AUC	0.885	0.035	0.850	0.845	0.015	0.830
Brier score	0.091	− 0.014	0.105	0.108	− 0.005	0.114

*LASSO* least absolute shrinkage and selection operator, *AUC* area under the curve

## Discussion

This research used comprehensive data recorded in MIMIC-III to develop two complete models by using methods of stepwise logistic regression and logistic regression with LASSO. These two new models were verified to have good predictive abilities for 30-day mortality of DVT patients (AUC both > 0.8 in the original data and after internal validation), good calibration (Brier scores were 0.091 and 0.108 in original data set, 0.105 and 0.114 after internal validation) and improvement in predictive abilities compared with widely-used SAPSII score (NRI [categorical and continuous] both > 0; IDI both > 0; *P* both < 0.05). The model obtained by using logistic regression with LASSO only included 8 variables, which was more suitable for rapid decision-making in ICU and was used to make the final nomogram.

DVT is a common and critical disease in ICU, which not only increases the time of bed-staying, but also endangers patients’ lives [22, 23]. And since the critically ill patients in ICU are usually complicated with a variety of basic diseases, such as cancer and multiple organ dysfunction (organ dysfunction accounts for 54.6% of the total DVT patients, metastatic cancer accounts for 11.0% and solid tumor accounts for 5.2% in this study), anticoagulation or thrombolysis therapy may cause major bleeding and even death [2]. The condition of DVT patients in ICU brings difficulties and challenges for doctors, especially for non-cardiovascular surgeons. A good scoring system or nomogram can help doctors in ICU predict the risk of mortality of DVT patients so as to strengthen monitoring or change decision-making. However, there is still lack of a prediction model specifically built for DVT patients in ICU, and none of previous studies had evaluated the predictive abilities of SAPSII, OASIS and SOFA on mortality of DVT patients in ICU. This study showed that the SAPSII score had better discrimination in predicting 30-day mortality in patients with DVT in ICU (AUC = 0.781 [95% CI 0.732–0.831]), while the other two scores showed worse abilities (AUC both < 0.7). This might be due to the reason that OASIS score doesn’t contain laboratory indicators and comorbidity information, while SOFA focuses more on organ failure. There are more than 15 indicators in SAPSII scoring model, which is more complicated to use compared with the nomogram developed in this study. The advantage of visualization and the categorical indicators of nomogram also increase the convenience of clinical application. Therefore, we recommend that doctors refer to our nomogram to quickly and accurately assess the 30-day risk of mortality in patients with DVT in ICU.

Old age can lead to a poor prognosis in patients with DVT in ICU, which may be due to a high risk of bleeding, cardiopulmonary circulatory disorders, or decreased immunity and more susceptibility to infection [24]. Age is also a risk factor that is included in SAPSII and pulmonary embolism severity index (PESI) which is suitable for predicting mortality in patients with PE [25]. Since development of model is based on a specific population or disease, there will be differences in the classification criteria in different models. In this study, we chose 75 years old as the best classification cut-off value according to the analysis of ROC curve, which is different from SAPSII and PESI. Similarly, as for systolic blood pressure, different diseases have different classification criteria. Low systolic blood pressure and high systolic blood pressure are usually associated with poor clinical prognosis [26–28]. More attention should be paid to the harm caused by low systolic blood pressure in ICU because patients are in critical state and may be complicated with heart failure or other organ dysfunction. This study used 110 mmHg as the truncated standard of systolic blood pressure, which was different from PESI.

The nomogram included sepsis and metastatic cancer among the comorbidity information of patients, and metastatic cancer had the highest score of all variables. Sepsis is a common disease in ICU which is also related with high mortality [29]. From the perspective of Virchow triad, systemic bacterial infection and inflammation, as well as damage to endothelial cells are important factors in promoting thrombosis. Many studies have pointed out that patients with sepsis were prone to secondary thrombotic diseases [30, 31], which might increase the risk of acute PE and death or difficulty in anticoagulation therapy. Metastatic cancer is also an important predictor in SAPSII score. In this study, metastatic cancer accounted for 11.0% of the total DVT patients, and was significantly correlated with clinical outcomes in both univariate and multivariate analyses (*P* = 0.001 in univariate analyses, *P* < 0.001 in two multivariate models). Cancer cells can differentially express genes including rat sarcoma family, phosphatase and tensin homolog, and tumor protein p53 to influence the genesis of thrombosis, which makes cancer patients themselves hypercoagulable [32, 33]. And cancer patients often have central venous catheterization because they are in need of long-term infusion [34], which also damages the endothelial and increases the risk of thrombosis. However, patients with metastatic cancer complicated with DVT have an increased risk of major bleeding during anticoagulation or thrombosis therapy [35, 36], which undoubtedly makes it difficult to treat this kind of patients. Whether inside or outside ICU, concerns about bleeding risk and unknown thrombosis are great barriers and challenges for thrombolytic therapy for DVT [37]. The nomogram proposed in this study can also assist doctors in risk grading or treating of DVT patients complicated with sepsis or cancer in ICU.

Laboratory indicators can provide doctors with a reference to the condition and help judge the prognosis of patients. Nomogram in this study included 4 indicators: blood urea nitrogen, albumin, lactate and NLR. In previous studies, RDW and anion gap were considered to be related to the prognosis of ICU patients [38, 39]. However, these two variables were screened out in logistic regression with LASSO and they were not shown in the nomogram in this study. Blood urea nitrogen was included in our nomogram and also in SAPSII. Blood urea nitrogen is considered to be a key factor reflecting the complex relationship among nutritional status, protein metabolism and renal condition. In several indicators (glomerular filtration rate, creatine and blood urea nitrogen) that reflect the renal function, blood urea nitrogen is more sensitive in reflecting the status of heart failure [40]. A previous retrospective study showed that high blood urea nitrogen was related with poor prognosis in critically ill patients in ICU. And even after adjusted for renal failure, blood urea nitrogen was still an independent variable to predict poor prognosis [41]. This study also showed that blood urea nitrogen could predict 30-day mortality independently in patients with DVT in ICU and 18.5 mg/dL was set as the cut-off value of classification. Albumin is an important component of human body, which has a variety of functions, such as extracellular antioxidants, buffers, immunomodulators and antidotes, and can alleviate inflammation caused by bacterial infection [42]. Previous studies showed that plasma albumin levels were reduced in many critically ill patients, up to 50% in patients with a value of lower than 35 g/L [43]. Decrease of albumin will cause disorder of blood osmotic pressure and serum composition, and may also be related with the loss of liver function, which will increase the mortality risk of DVT patients. In this study, the classification cut-off value of albumin was 3 g/dL. Many previous studies have pointed out the relationship between high lactate content and high mortality or poor prognosis [44, 45]. In this study, lactate could be used as an independent variable to predict the clinical outcome as well, classification cut-off value of which was set as 2.25 mmol/L. NLR is an inflammatory indicator that can reflect the level of inflammation, which was also an important risk factor for predicting the clinical outcome in this study. Inflammation not only can promote thrombosis, but also has an important effect on the progression of DVT patients in ICU [46]. The classification cut-off value of NLR was set as 12.6.

Our research has several strengths. Firstly, our study is the first to develop a 30-day mortality risk score for DVT patients in ICU. Secondly, we used two methods to develop two complete models respectively and tested their predictive abilities in original training set and internal validation. Among them, LASSO belongs to machine learning and can simplify the model while ensuring the predictive ability of the model, which provides methodological convenience for us to make models suitable for clinical application. Thirdly, our final model for clinical application was presented as nomogram and all the included continuous variables were classified as categorical variables according to certain criteria, which provides great convenience for clinicians.

Our research also has limitations. Firstly, the missing data of albumin and lactate contained in the nomogram were both more than 20% before imputation, which may have an impact on the predictive ability of the model in external validation. Secondly, our study lacks data of blood gas analysis, for the reason that we found too much data missing in blood gas analysis in initial inclusion of variables. For the sake of the stability of the model, we cleaned out most of the variables of blood gas analysis though there were many variables that we considered to be excellent in predicting the clinical outcomes. Further studies can incorporate some items of blood gas analysis on the basis of our model to explore the predictive abilities of blood gas analysis on the mortality of patients with DVT. Thirdly, the clinical outcome we set was all-cause mortality rather than death directly related to DVT or embolic death (or PE), which was due to recording limitations of open database. And we can’t have access to the patient's clinical end point and do further follow up.

## Conclusions

Using the data from the open database MIMIC-III, we developed two sets of prediction models for 30-day mortality risk of DVT patients in ICU with two statistical methods, and selected the more concise model to make a nomogram for clinical application. The prediction ability of the nomogram is better than traditional scores, and can be used in the decision-making of DVT which has a high prevalence in ICU.

## Supplementary Information

**Additional file 1.** Classification of variables.

## Data Availability

The datasets generated and/or analysed during the current study are available in the MIMIC-III critical care database, which can be accessed on https://mimic.physionet.org/ [16].

## Abbreviations

ICU: Intensive care unit
ICD: International classification of diseases
DVT: Deep vein thrombosis
VTE: Venous thromboembolism
PE: Pulmonary embolism
MIMIC-III: Medical Information Mart for Intensive Care III
LASSO: Least absolute shrinkage and selection operator
ROC: Receiver operating characteristic
AIC: Akaike information criterion
AUC: Area under the curve
NRI: Net reclassification improvement
IDI: Integrated discrimination improvement
SAPSII: Simplified acute physiology score II
OASIS: Oxford acute severity of illness score
SOFA: Sequential organ failure assessment
PESI: Pulmonary embolism severity index
spO2: Percutaneous oxygen saturation
RDW: Red blood cell volume distribution width
PTT: Partial thromboplastin time
PT: Prothrombin time
NLR: Neutrophil-to-lymphocyte ratio
LMR: Lymphocyte-to-monocyte ratio
SD: Standard deviation
SE: Standard error
CI: Confidence intervals
