# Supplementary material for: Prognostic nomogram for 30-day mortality of deep vein thrombosis patients in intensive care unit
Source: BMC Cardiovasc Disord. 2021 Jan 6;21:11. doi: 10.1186/s12872-020-01823-4 (PMC7788873; doi:10.1186/s12872-020-01823-4)
Supplement: Supplementary file 1 — Additional file 1. Classification of variables. [file 12872_2020_1823_MOESM1_ESM.docx]

Table S1. Classification of variables.

|  | Variables | Classifications |
| --- | --- | --- |
| General Condition |  |  |
|  | age | <75 and ≥ 75 |
|  | heartrate (bpm) | ≤80 and 80~110 and ≥110 |
|  | systolic blood pressure (mmHg) | <110 and ≥ 110 |
|  | diastolic blood pressure (mmHg) | <62.5 and ≥62.5 |
|  | respiratory rate (1/min) | <30 and ≥30 |
|  | temperature (℃) | <36 and ≥36 |
|  | spO2 (%) | ≤97 and 97~98 and ≥98 |
|  | glucose (mg/dl) | <140 and ≥140 |
| laboratory indicators |  |  |
|  | 50-RDW (%) | <34.5 and ≥34.5 |
|  | lymphocyte (%) | <8.5 and ≥8.5 |
|  | log10(monocyte (%)) | None ^a^ |
|  | neutrophil (%) | ≤80 and 80~90 and ≥90 |
|  | hematocrit (%) | <32.5 and ≥32.5 |
|  | white blood cell (K/uL) | <14 and ≥14 |
|  | platelet count (K/uL) | ≤200 and 200~300 and ≥300 |
|  | potassium (mEq/L) | ≤4 and 4~5 and ≥5 |
|  | sodium (mEq/L) | ≤135 and 135~145 and ≥145 |
|  | anion gap (mEq/L) | ≤14 and 14~16 and ≥16 |
|  | bicarbonate (mEq/L) | ≤23 and 23~27.5 and ≥27.5 |
|  | chloride (mEq/L) | ≤100 and 100~105 and ≥105 |
|  | blood urea nitrogen (mg/dL) | <18.5 and ≥18.5 |
|  | log10(creatinine (mg/dL)) | ≤-0.1 and -0.1~0.2 and ≥0.2 |
|  | albumin (g/dL) | <3 and ≥3 |
|  | hemoglobin (g/dL) | <11 and ≥11 |
|  | log10(lactate (mmol/L)) | < log10(2.25) and ≥ log10(2.25) |
|  | PTT (sec) | ≤50 and 50~75 and ≥75 |
|  | PT (sec) | <14.5 and ≥14.5 |
|  | log10(NLR) | <1.1 and ≥1.1 |
|  | log10(LMR) | <0.4 and ≥0.4 |

Abbreviations: spO2, percutaneous oxygen saturation; RDW, red blood cell volume distribution width; PTT, partial thromboplastin time; PT, prothrombin time; NLR, neutrophil-to-lymphocyte ratio; LMR, lymphocyte-to-monocyte ratio.

^a^Monocyte was removed from the selected variables because of the poor prediction of the outcome.
